# Determinants of unequal HIV care access among people living with HIV in Peru

**DOI:** 10.1186/1744-8603-9-22

**Published:** 2013-05-17

**Authors:** Alfonso Silva-Santisteban, Eddy R Segura, Clara Sandoval, Maziel Girón, Margarita Petrera, Carlos F Caceres

**Affiliations:** 1 Unit of Health, Sexuality and Human Development, Cayetano Heredia University School of Public Health, Lima, Peru. Av. Armendariz 445, Lima 18, Peru

**Keywords:** Health care access, Equity, HIV, Antiretroviral treatment, Transgender, Social determinants of health, Peru

## Abstract

**Background:**

Equity in access to health care among people living with HIV (PLHA) has not been extensively studied in Peru despite the fact there is significant social diversity within this group. We aimed to assess the extent to which health care provision to PLHA, including ARVT, was equitable and, if appropriate, identify factors associated with lower access.

**Methods:**

We conducted a survey among adult PLHA in four cities in Peru, recruited through respondent-driven sampling (RDS), to collect information on socio-demographic characteristics, social network size, household welfare, economic activity, use of HIV-related services including ARV treatment, and health-related out-of-pocket expenses.

**Results:**

Between September 2008 and January 2009, 863 individuals from PLHA organizations in four cities of Peru were enrolled. Median age was 35 (IQR = 29–41), and mostly male (62%). Overall, 25% reported to be gay, 11% bisexual and 3% transgender. Most PLHA (96%) reported access to some kind of HIV-related health service, and 84% were receiving those services at a public facility. Approximately 85% of those reporting access to care were receiving antiretroviral treatment (ARV), and 17% of those not in treatment already had indication to start treatment. Among those currently on ARV, 36% percent reported out-of-pocket expenses within the last month. Transgender identity and age younger than 35 years old, were associated with lower access to health care.

**Conclusions:**

Our findings contribute to a better social and demographic characterization of the situation of PLHAs, their access to HIV care and their source of care, and provide an assessment of equity in access. In the long term, it is expected that HIV care access, as well as its social determinants, will impact on the morbidity and mortality rates among those affected by the HIV/AIDS epidemic. HIV care providers and program managers should further characterize the barriers to healthcare access and develop strategies to resolve them by means of policy change, for the benefit of the health service users and as part of the national response to the HIV/AIDS epidemic within a human rights framework.

## Background

The concept of social determinants of health (SDH) has been defined broadly as encompassing the full set of social conditions in which people grow, live, work and age [1]. SDH operate at different causal levels such as socioeconomic context, exposure, vulnerability, access to health care, health outcomes and consequences, and can be identified and addressed at those levels [2]. However, despite this need, there is a lack of appropriate SDH and disease outcome analyses to quantify the contribution of SDH variables to specific outcomes of interest, such as health access [3].

In this line of thinking, since early in the epidemic, the HIV field has been enriched by the analysis of social determinants of HIV acquisition and disease progression, usually showing that HIV incidence and disease outcomes are related to the social position an individual occupies in the social gradient [4]. Monitoring the prevalence of those factors and their individual contributions to health outcomes has proved important in understanding not only disease incidence and critical preventive interventions, but also access to appropriate care and treatment, and ways in which those can be improved [5]. Unfortunately, as current evidence supports, there is a paucity of studies on the SDH of access and adherence to antiretroviral therapy, especially in Latin American countries [6].

After the contrasting conditions of access to HIV treatment between the global North and the global South became more evident towards the end of the 1990’s, key initiatives were taken to improve HIV care access in lower and middle-income countries. In June 2001, the United Nations General Assembly Special Session on HIV (UNGASS) set timeline goals to be reached according to commitments among governments, civil society and the private sector across the nations involved [7]. Those are based on the HIV Declaration of Commitment (DoC) on HIV/AIDS adopted by all United Nations member states. Recent evidence indicates continued progress in scaling up antiretroviral treatment, with global coverage increasing by 36 per cent in 2008 alone [8].

Over the past 25 years, the government of Peru, organized civil society groups, and academic institutions, have developed multiple collaborative efforts to fight the HIV/AIDS epidemic [9,10]. Those efforts were boosted in the past 8 years with support from the Global Fund to fight AIDS, Tuberculosis and Malaria (GFATM) that, among other things, helped to launch the National Antiretroviral Treatment Program which started free-of-cost antiretroviral treatment provision at government-administered health facilities [9,11]. Currently, the existing Peruvian health care providers, Ministry of Health (MoH), Social Security (ESSALUD), HIV Committee for Uniformed Institutions; and private sector systems, offer specialized service to people living with HIV/AIDS (PLHA) including: diagnosis, counseling, prevention and prophylaxis of opportunistic infections; and antiretroviral treatment (ARVT), including clinical, viral and immunological monitoring prior to and during such treatment. Most existing services were initially implemented as part of the activities sponsored by GFATM, but most of them are now government-funded [12]. However, the sole existence of such health services does not necessarily warrant their success. Current evidence shows that, in 2009, 12,473 PLHA were treated at MoH centers, covering an estimated 75% of clients registered in the National HIV Care Program [11]. This figure did not take into account PLHA receiving care from other health providers.

As part of the evaluation of HIV projects implemented under Global Fund support, a few studies have focused on access to HIV treatment and care among PLHA in Peru [13-16]. Those studies, however, were rapid assessments among clients recruited in HIV care facilities; hence potentially overestimating treatment coverage. Nevertheless, without a sampling framework for this population, particularly in the context of stigma, better sampling schemes were not available limited. In recent years methods such as respondent-driven sampling (RDS), based on social networks, have become increasingly popular to reach hidden populations [17]. While factors linking other hidden social networks (e.g. social and sexual interaction among men who have sex with men, socialization and equipment sharing among injection drug users) are not necessarily present among PLHA and some of them may in fact prefer isolation due to fear of rejection and abandonment [18], since early in the Peruvian epidemic self-support groups and community-based organizations promoted networking. On that basis, we used RDS to assemble a sample of PLHA from five cities in Peru, with the goal of assessing HIV-related health care access, and exploring the relationship between such access and key socio-demographic and socio-economic determinants. Additionally, we explored the relationship between entitlement to specific sources of health care and effective utilization of those sources for HIV care.

## Methods

### Study design, enrollment

A population-based cross-sectional study targeting individuals 18 years or older with a known HIV diagnosis was conducted in 5 Peruvian cities: Lima and Callao (considered as a single metropolitan area), Chiclayo (in the Northern coast), Arequipa (in the highlands) and Iquitos (in Amazonia) between January and July 2009. Participants were reached and recruited through respondent-driven sampling (RDS), which has been extensively used for biological and behavioral surveillance in hard-to-reach populations [17,19]. Briefly, an initial group of 7 “seeds”, who were members of PLHA organizations in each of those 4 metropolitan areas were invited to participate and, if accepting, were instructed to refer other PLHAs they knew, who might or might not be affiliated to their organizations. Selected seeds belonged to PLHA organizations in each of those 4 metropolitan areas. In turn, each referred participant was asked to invite three other PLHA of their personal social networks, repeating this referral cycle as many times as needed until reaching the desired sample size. Eligibility criteria included having a known HIV diagnosis, being older than 18 years old; having a study coupon; and being able to provide written informed consent.

Effective access to any type of HIV-related health service was the primary outcome. It was defined as at least one office visit, or program enrollment reported by participant in any of the HIV health services available in Peru (public, social security, armed forces services and private services) in the last 12 months, and it was operationalized as a binary variable (i.e. access vs. no access).

### Sample size

Sample size was calculated using the Epi Info 6.0v package (CDC, Atlanta, GA). As no previous data on health care access among PLHA was available in the country, we used a conservative approach estimating access in 50%, onsidering a margin of error of ± 5%; and a design effect = 2 [20]. A total sample size of 800 individuals was calculated.

### Study variables, data collection

The survey explored socio-demographic characteristics including age, sexual and gender identity and education). Household welfare (i.e. classifying households in socio-economic strata) was measured through a scale developed by the Ministry of Finance (National Household Targeting System - SISFOH scale) to identify households in poverty and extreme poverty, and define targets for social programs. Members’ characteristics, overcrowding and possession of specific goods (e.g. home appliances) were assigned a specific weight, so that the sum of all weighted criteria determined the final household welfare status indicator. Specific socio-economic classifications for each city of the study were generated using differential weights, in order to account for regional variations as proposed by the Peruvian Ministry of Finance [21]. Economic activity, general expenses, use of HIV-related health services including ARV treatment, health-related out-of-pocket expenses, and participation in GFATM-sponsored microenterprise projects (specific interventions promoted by the GFATM programs to improve the living conditions of PLHA). This study did not focus on clinical aspects of the management of HIV, since those were beyond its scope.

A previous version of the instrument had been used in other studies targeting a similar population at health facilities [13,22].

### Study procedures

Study staff explained the study process to eligible participants and obtained written consent before the face-to-face interview. Participants who completed the interview received S/. 20(approximately US $7). Participants were invited to recruit three acquaintances living with HIV, and older than 18 years. As incentive, for each referred person who was successfully recruited each participant received a raffle ticket for a scholarship to participate in a six-month technical course, to be selected by the participant that won the raffle.

### Data analysis

Central tendency and dispersion measures [mean, standard deviation (SD), median, and interquartile range (IQR)] were computed for continuous variables (age and out-of-pocket expenses) to describe the study population, and absolute and relative frequencies were computed for categorical, nominal and ordinal variables. Naïve and RDS-adjusted estimates were computed. For the latter, we used the inverse of the individual’s reported social network size as weight to compute RDS-adjusted estimates (approach known as RDS II estimation) [23,24]. This approach enables to weigh down the contribution of participants who belong to bigger personal networks, and thus are more likely to be enrolled in the study, and on the contrary weighs up the contribution of those with smaller social networks. It does not take into account homophily (i.e the fact that people with a certain characteristic recruit their equals), as the traditional method does (i.e. approach known as RDS I estimation) [25].

To test the association between the hypothesized predictors and access to any type of HIV-related health service, we used the chi square test (or Fisher exact test when appropriate). Predictor variables under study included: biological sex, sexual/gender identity, age (dichotomized as ≤ 35 and > 35 years old), geographic region, educational attainment, marital status, being the head of a household, insurance and type of insurance, employment, and household welfare. For all statistical analyses, a p-value < 0.05 was considered to reject the null hypothesis and claim statistically significant differences. All analyses were conducted using the STATA v10 software.

Tables of results show both crude estimates and corrected RDS II estimates (as indicated in table headings).

### Regulatory and ethical aspects

Ethical approval for the overall study execution, its protocol and instruments was granted by the Cayetano Heredia University Institutional Review Board (Lima, Peru). After volunteering to enroll, all potential participants received a detailed explanation about the study goals, procedures and associated risks. Those who were willing to participate signed the informed consent form. After this, trained field staff used structured questionnaires to interview the participants. Interviews were conducted in private environments used solely for the purpose of the interview, ensuring privacy and confidentiality.

## Findings

Between September 2008 and January 2009, 863 individuals who were members of the social networks of initial recruiters (seeds) from PLHA organizations in the cities of Lima/Callao, Chiclayo, Arequipa and Iquitos, were enrolled.

### Characteristics of the study population

As shown in Table 1, the study population (N = 863) was mainly composed by young adults (age mean = 35 years, standard deviation = 9, age median = 35, interquartile range = 29–41, range = 18–62), and mostly male (62%). Self-reported sexual identity was: 25% gay, 11% bisexual, 3% transgender and 60% heterosexual. 56% came from Lima-Callao, 15% from Chiclayo, 16% from Arequipa and 13% from Iquitos. Thirty-one percent had completed elementary school, 34% had completed high school and 34% had at least some higher education. The majority of the population (57%) was single, followed by those living with a domestic partner (21%), and married (9%). Of those currently working (61%), only 18% had a job that included social benefits. Median of monthly income was S/.550 (About USD 200), and IQR was S/. 300 to 800 (about 100.00 to 300.00 USD). Of those not currently working, 38% had searched for a job in the past 7 days.

**Table 1:** Socio-demographic characteristics of PLHA participants, Peru, 2008 (N = 863)

**Characteristics and categories**	**Number (Percentage)**	**RDS II (%)**
Sex		
Male	533 (61.8%)	63.1
Female	330 (38.2%)	36.9
Identity (self-assigned)		
Gay	217 (25.1%)	20.9
Transgender	29 (3.4%)	3.4
Bisexual	96 (11.1%)	11.2
Heterosexual	519 (60.1%)	64.0
Lesbian	2 (0.3%)	0.6
Age		
18–35 years old	464 (53.8%)	58.6
Older than 35 years old	399 (46.2%)	41.4
Range	18–62	
Median and interquartile range	35 (28–41)	
Mean and standard deviation	35 (9)	
Geographic area		
Lima & Callao (Coast)	481 (55.7%)	30.9
Lambayeque (Northern Coast)	134 (15.5%)	15.6
Arequipa (Southern Highlands)	135 (15.7%)	27.5
Iquitos (Amazonia)	113 (13.1%)	26.0
Educational attainment		
None to some Elementary School	47 (5.5%)	7.7
Full Elementary School to some High School	224 (26.0%)	32.8
Full High School	295 (34.1%)	28.8
University or Technical	297 (34.4%)	30.7
Marital status		
Single	491 (56.9%)	52.7
Married	75 (8.7%)	11.9
Domestic partner	178 (20.6%)	20.4
Divorced	64 (7.4%)	8.3
Widowed	55 (6.4%)	6.8
Year of HIV diagnosis		
Before 2000	89 (10.3%)	6.5
2000–2002	117 (13.6%)	9.7
2003–2005	250 (29.0%)	22.2
2006–2008	407 (47.1%)	61.6

Regarding health care entitlements, 30% were entitled to care paid by the limited-liability, government-sponsored SIS (Comprehensive Health Insurance, mainly offered to people in extreme poverty and for maternal and child care), 16% had Social Security affiliation (i.e. “EsSalud”, linked to a longer-standing health provision system for formal employees), 3% were entitled to other systems, and 52% had no insurance at all. In the overall study population, 62% were diagnosed with HIV infection between the years 2006–2008, 22% between 2003–2005, 10% between 2000–2002, and 6% before the year 2000 (see Table 1). According to the official scale for classification of household welfare, 1% of the study population lived in extreme poverty, 20% in poverty, and 78% were not poor (see Table 2).

**Table 2:** Household welfare distribution of PLH by geographic area, Peru, 2008 (N = 863)

**Poverty level**	**Household welfare indicator (SISFOH)**	**Coast (Lima, Callao, and Chiclayo) crude estimate**	**Coast adjusted estimate (RDS-II)**	**Andes (Arequipa) crude estimate**	**Andes adjusted estimate (RDS-II)**	**Amazonia (Iquitos) crude estimate**	**Amazonia adjusted estimate (RDS-II)**
Extreme poverty	SISFOH1	0 (0.0%)	0	0 (0.0%)	0	0 (0.0%)	0
	SISFOH2	0 (0.0%)	0	0 (0.0%)	0	8 (7.1%)	6.3
Not extreme poverty (Moderate poverty)	SISFOH3	16 (2.6%)	5.3	7 (5.2%)	9.1	8 (7.1%)	5.9
	SISFOH4	28 (4.5%)	5.8	9 (6.7%)	6.3	19 (16.8%)	16.1
	SISFOH5	51 (8.3%)	13.2	23 (17.0%)	13.7	20 (17.7%)	17.5
Total below poverty line		95 (15.4)	24.3	39 (28.9)	29.1	47 (48.7)	45.8
Not in poverty	SISFOH6	76 (12.4%)	16.5	15 (11.1%)	11.8	12 (10.6%)	10.8
	SISFOH7	444 (72.2%)	59.2	81 (60.0%)	59.1	46 (40.7%)	43.4
Total above poverty line		520 (84.6)	75.7	96 (71.1)	70.9	58 (51.3)	54.2

### Access to HIV care

Most (96%) of PLHA participating in the study reported they had access to some kind of HIV-related health service, and 84% that they were receiving those services from a MoH facility (see Table 3). Approximately 77% of those reporting access to care were receiving ARV treatment, and 22% of those who were not in treatment already had indication to start treatment. 27% percent of those currently on ARV treatment reported out-of-pocket expenses within the last month. Treatment coverage varied according to year of diagnosis: of those diagnosed before 2000, 97% were receiving ARV treatment, while figures were 92%, 92% and 76% for participants diagnosed between 2000–2002, 2003–2005, and 2006–2008, respectively.

**Table 3:** Characteristics of health care access and out-of-pocket expenses reported by PLHA participants, Peru, 2008 (N = 863)

**Characteristics and categories**	**Crude estimate**	**Adjusted estimate (RDS-II)**
Currently receiving any HIV-related health service (medical check-ups, laboratory testing, or ARV treatment) (N = 863)	832 (96.4%)	95.6%
Institution/Program were PLHA participant receives HIV care (N = 827/832)		
Public (Ministry of Health) Hospital	653 (79.0%)	82.5%
Public Non-Hospital Health facility	26 (3.1%)	1.5%
Social Security Health facility (EsSalud)	73 (8.8%)	9.0%
Police and Armed Forces Health facility	14 (1.7%)	2.2%
Non-governmental organizations	52 (6.3%)	4.3%
Religious institutions	9 (1.1%)	0.5%
Currently receiving ARV treatment (N = 830/832)	703 (84.7%)	76.8%
Year ARV treatment was started (N = 700/703)		
Before 2000	12 (1.7%)	1.7%
2000–2002	36 (5.2%)	3.1%
2003–2005	234 (33.4%)	22.9%
2006–2008	418 (59.7%)	72.3%
If not currently receiving ARV, PLHA received indication to start ARV (N = 123/127)	21 (17.1%)	21.6%
If currently receiving ARV treatment, any reported HIV-related out-of-pocket expenses within the last month (N = 697/703)	238 (36.0%)	26.7%
Reported HIV-related out-of-pocket expenses within last month (N = 218/238) (Median, range and interquartile range, in nuevos soles)	50 (8 – 7,500; 25–80).	
If currently receiving ARV treatment, any reported out-of-pocket expenses for laboratory testing (Only those who started ARV between 2006–2008, N = 417/418)	411 (98.6%)	99.1%
Reported out-of-pocket expenses for laboratory testing (Only those who started ARV between 2006–2008, N = 297/418) (Median, range and interquartile range, in nuevos soles)	100 (10–3000; 60–80)	
Any reported not HIV-related out-of-pocket expenses within the last month for office visits, laboratory testing or medications not prescribed for HIV/AIDS (N = 863)	267 (30.9%)	27.1%
Reported not HIV-related out-of-pocket expenses within the last month (N = 246/267) (Median, range and interquartile range, in nuevos soles)	50 (4–2500; 25–150)	

Table 4 shows the results of bivariate analysis of heath care access and hypothesized determinants. Age younger than 35 and transgender (male to female) identity were associated with lower access to care (p <0.05).

**Table 4:** Lack of HIV-related health care access according to key characteristics of PLHA participants, Peru, 2008 (N = 863)

**Characteristics and categories**		**Crude estimate**	**Adjusted estimate (RDS-II)**	**P value**
Biological sex	Male	22 (4.1%)	5.2	0.30
	Female	8 (2.7%)	2.9	
Sexual/Gender Identity	Homosexual/Gay	5 (2.3%)	2.7	<0.05
	Transgender	6 (20.7%)	27.3	
	Bisexual	3 (3.1%)	2.7	
	Heterosexual	17 (3.2%)	3.8	
Age Range	35 years old or younger	24 (5.2%)	6.2	<0.05
	Older than 35 years old	7 (1.7%)	1.7	
Geographic area	Coast (Lima-Callao and Chiclayo)	20 (3.2%)	3.0	0.27
	Andes (Arequipa)	7 (5.2%)	4.7	
	Amazonia (Iquitos)	4 (3.5%)	0.9	
Educational attainment	None/Some Elementary School	0 (0.0%)	0	0.33
	Completed elementary school	11 (4.9%)	7.1	
	Completed High School	10 (3.4%)	3.2	
	Some higher education	10 (3.4%)	3.6	
Marital status	Single	21 (4.3%)	4.5	0.57
	Married	2 (2.7%)	8.3	
	Domestic partner	5 (2.8%)	3.6	
	Divorced/Separated	2 (3.1%)	1.5	
	Widowed	1 (1.8%)	1.9	
Head of household	Yes	12 (3.6%)	4.4	0.96
	No	19 (3.6%)	4.3	
Health insurance	Social Security, Forces, Private	4 (2.7%)	1.9	0.80
	SIS (Ministry of Health)	7 (3.2%)	5.4	
	No insurance	20 (4.0%)	4.6	
Currently working	Yes	18 (3.4%)	5.1	0.37
	No	13 (3.8%)	3.3	
Poverty	Extreme poverty	0 (0.0%)	0	0.13
	Moderate poverty	14 (7.7%)	8.0	
	Not in poverty	17 (2.5%)	3.0	

### Coverage by health insurance and source of HIV care

The distribution of participants according to their entitlement to health provision systems and the current provider of HIV care services is shown in Table 5. Approximately 40% of interviewees covered by health insurance other than the Ministry of Health (i.e. Social security, Police and armed forces, and private providers) received HIV services at MoH facilities. This group represented 8% (51/679) of all individuals in the sample who received HIV care at the MoH.

**Table 5:** PLHA participants distributed according to Institution or Program where HIV services are received and type of health insurance, Peru, 2008 (N = 827/832)

**Entitlement to specific health provision regimes among People living with HIV**	**Institution or Program where PLHA participants receive HIV care**					
	**Social Security, Police and Armed Forces, and Private**		**Facilities of the Ministry of Health**		**Other institutions (Religious groups and NGOs)**	
	**Crude estimate**	**Adjusted estimate (RDS-II)**	**Crude estimate**	**Adjusted estimate (RDS-II)**	**Crude estimate**	**Adjusted estimate (RDS-II)**
Social Security, Police and Armed Forces, and Private	84 (57.9%)	56.6%	51 (35.2%)	40.0%	10 (6.9%)	3.4%
Ministry of Health (SIS)	0 (0.0%)	0.0%	196 (92.9%)	95.7%	15 (7.1%)	4.3%
No insurance	3 (0.6%)	1.5%	432 (91.7%)	93.0%	36 (7.7%)	5.5%

## Discussion

In this study, health care access (as reported by participants) was assessed through estimating the proportion of the population receiving HIV health care from any type of provider and came to be close to 96% across 4 Peruvian cities. This high coverage might be explained by the fact that ARVT provision is free of cost as part of public policy of the Peruvian Ministry of Health. People with an HIV diagnosis who want to receive treatment and care at public hospitals, or at local NGOs that have received accreditation from the Ministry of Health, have to be enrolled in the ARVT program for follow-up in order to start on medication promptly when needed. The National Social Security System (ESSALUD) provides services to those on an employment payroll and private services for people that will pay for them. All public and private health services have to comply with standards established by the Ministry of Health. As results show, the majority of people receive treatment and care from public hospitals (83%), followed by social security health facilities (9%). An initial need to reach a somewhat overestimated coverage target in 2005–2006, led the MoH to actively search for cases [12]. It also could be hypothesized that, since the establishment of the ARVT Program as a free-of-cost strategy, access to health services may have increased among PLHA in order to receive ARVT when needed.

Lower access to HIV/AIDS services was associated with transgender (male to female) identity and being younger than 35 years old. A previous study from our group confirmed transgender people as the most vulnerable population to HIV in Peru, with a prevalence of 30%, more than doubling the figures observed among MSM [26]. This population experiences very specific conditions of exclusion and marginalization that partly explain their vulnerability not only by means of lower access to health care but also to education and justice. This is supported by studies that show that transgender women report more difficulties when dealing with health providers than men who have sex with men and sex workers [12]. Despite progress in quality of care for transgender people, stigma around both HIV and gender identity, together with concerns about confidentiality (i.e. being seen by their peers, particularly if involved in sex work), or difficulties in keeping up with medical prescriptions and treatment pick-up times, play a role in both lower access and treatment attrition [27]. These key elements should be taken into account to resolve this serious access issue.

The association between age younger than 35 y.o. and lower access is less clear and requires further study. Likely explanations are, however, the well-known resistance of younger people to use health care, and issues regarding education and income [28], where potentially lower economic resources to afford out-of-pocket expenses could act as a barrier to start HIV care. Likewise, issues such as lower family responsibilities and potentially a better health status (i.e. earlier infection stages) might explain this difference. Further explanation of this finding should guide specific interventions to improve access in this age group.

The majority of PLHA who report access to HIV care in the study are already receiving ARVT. Since all these people are already being seen by health providers the estimate becomes a measure of Program effectiveness. The estimate is similar to the figure found in a survey carried out with PLHA in health facilities across several Peruvian cities in 2008 [15], and this consistency could be interpreted as a performance parameter of the Peruvian HAART Program (i.e. approximately a fifth of PLHA registered in the Program and with ARV indication are waiting to start therapy at a given point in time). Since numbers were small, it becomes difficult to further explore whether certain groups of PLHA were more likely to experience delayed access to treatment, and such issue remains a topic for further research. The delay might be explained by a shortage of medication supply in health facilities [29], or by a delay due to other steps in the process, such as completion of all laboratory testing needed, which in many hospitals is financed out-of-pocket. Shortage of ARV medication for PLHA has been described as one of the major problems encountered by users of HIV/AIDS public services [30] and is one of the most important challenges the public sector has to face in the next years. Additionally the national guidelines for ARV provision, updated in July 2012 in line with WHO recommendations [31], indicate that ARVT should be provided to all people living with HIV with CD4 counts below 350 cells/mm^3^, while the previous cut-off point was CD4 < 200. This change will necessarily increase the number of people with indication of treatment. The fact that 76% of those diagnosed with HIV between 2006 and 2008 were receiving ARV therapy by the time the study was conducted (2009) could indicate a high proportion of late HIV diagnosis, although this topic was not explored in our survey and could not be corroborated with official information from the public health system. Nonetheless, there is evidence of a sustained decrease in HIV mortality since the start of the National HAART Program [32].

An assessment of effective HIV-care provider vs. general health care provider entitlement showed that one third of PLHA covered by non-public health insurance used MoH HIV services (which are in fact open to all). In turn, they represent almost one tenth of MoH HIV care recipients. This unnoticed and silent transfer of users from various programs to the public system is evidence of a cross-subsidy. In part, such transfer was even promoted by the MoH at the time of inception of the treatment program, when it was still funded by the GFATM and the targets set up were too high, as a result of inaccurate estimation of the number of potential users [9,33]. At this point, however, specific health insurance systems should pay for the ARV treatment of PLHA who are entitled to their services, and leave the public system for those not covered. Additionally, enrollment in ARVT should be friendly and simple regardless of the provider. For example, accessing ARVT through the Social Security system is still more complex since a small co-payment by employers and time-consuming paperwork by the patients are still needed.

Poverty has been described as a barrier because of its impact on how people perceive and express needs, partly due to competing subsistence demands and cost barriers to accessing services [2,34,35]. Previous studies provide evidence of the relationship between poverty and health care access in Peru. In general, families reporting lower income and structural constraints (e.g. lack of basic services) have the lowest levels of health care access [36]. In other countries, the fact that PLHAs of lower socio-economic status are more likely to start ARVT later and exhibit shorter survival than PLHA of higher socio-economic status has already been recognized [37]. In our study, however, poverty was not associated with lower access to health services when we corrected for RDS II analysis (although it was in unadjusted estimates), in part probably because of the small numbers of those who lacked such access and the lower frequency of poverty. This potential relationship should be further explored, particularly given the HIV program’s investment in micro-enterprise interventions for economic empowerment of PLHA [16].

### Limitations

Among the limitations of this study, it is difficult to establish if PLHA form a social network based on the fact of being HIV positive. Some PLHA community groups exist where members interact among themselves, often across divisions of gender and sexual orientation, but this does not imply that, overall, every person with HIV will form a network and interact as a group. However, we used RDS in four metropolitan areas of Peru and succeeded in obtaining a broader sample, apparently more representative concerning PLHA gender composition, source of HIV/AIDS care, and sexual orientation, compared to other surveys [12-15]. Nevertheless, it is possible that this recruitment approach more efficiently reached networks of people who are closer to CBOs and activists who have more access to information and to health services, hence still over-estimating access among PLHA. Additionally, our limited focus on clinical indicators of the present and past health status of participants may have affected our understanding of other barriers to health care access, for example, advanced disease and inability to mobilize to seek care.

## Conclusion

This is the first study of access to care among PLHA in Peru using RDS, and also the first study conducted through a sampling approach other than convenience recruitment. On a sample of 863 PLHA from 4 cities, it found that most PLHA (96%) reported access to some kind of HIV-related health service, and 85% of them were already receiving ARVT, and only 17% of those not in treatment had an indication to start ARVT. Transgender identity and age younger than 35 years old, but not poverty, were associated with lower access to health care. Finally, 40% of interviewees covered by health insurance other than the Ministry of Health received HIV services at MoH facilities, and represented 8% of the population effectively receiving HIV care from the MoH.

Our findings contribute to a better social and demographic characterization of the situation of PLHAs, their access to HIV care and their source of care, and provide an assessment of equity in access. In the long term, it is expected that HIV care access, as well as its social determinants, will impact on the morbidity and mortality rates among those affected by the HIV/AIDS epidemic. HIV care providers and program managers should further characterize these barriers and develop strategies to resolve them by means of policy change, for the benefit of the health service users and as part of the national response to the HIV/AIDS epidemic within a human rights framework.

## Competing interests

The authors declared that they have no competing interest.

## Authors’ contributions

ASS contributed to study design, implementation, analysis (including the RDS approach) and writing. ERS contributed to study design, analysis and writing. CS contributed to writing of the first report. MG contributed to study design and implementation. MP contributed to the adaptation of the SES scale. CFC contributed to study design, analysis, write-up and final editing. All authors read and approved the final manuscript.
